# CD166/ALCAM Expression Is Characteristic of Tumorigenicity and Invasive and Migratory Activities of Pancreatic Cancer Cells

**DOI:** 10.1371/journal.pone.0107247

**Published:** 2014-09-15

**Authors:** Kenji Fujiwara, Kenoki Ohuchida, Masafumi Sada, Kohei Horioka, Charles D. Ulrich, Koji Shindo, Takao Ohtsuka, Shunichi Takahata, Kazuhiro Mizumoto, Yoshinao Oda, Masao Tanaka

**Affiliations:** 1 Department of Surgery and Oncology, Graduate School of Medical Sciences, Kyushu University, Fukuoka, Japan; 2 Department of Anatomic Pathology, Graduate School of Medical Sciences, Kyushu University, Fukuoka, Japan; 3 Research Fellow of the Japan Society for the Promotion of Science, Tokyo, Japan; 4 Kyushu University Hospital Cancer Center, Fukuoka, Japan; Roswell Park Cancer Institute, United States of America

## Abstract

**Background:**

CD166, also known as activated leukocyte cell adhesion molecule (ALCAM), is expressed by various cells in several tissues including cancer. However, the role of CD166 in malignant tumors is controversial, especially in pancreatic cancer. This study aimed to clarify the role and significance of CD166 expression in pancreatic cancer.

**Methods:**

We performed immunohistochemistry and flow cytometry to analyze the expression of CD166 in surgical pancreatic tissues and pancreatic cancer cell lines. The differences between isolated CD166+ and CD166- pancreatic cancer cells were analyzed by invasion and migration assays, and in mouse xenograft models. We also performed quantitative RT-PCR and microarray analyses to evaluate the expression levels of CD166 and related genes in cultured cells.

**Results:**

Immunohistochemistry revealed high expression of CD166 in pancreatic cancer tissues (12.2%; 12/98) compared with that in normal pancreas controls (0%; 0/17) (p = 0.0435). Flow cytometry indicated that CD166 was expressed in 33.8–70.2% of cells in surgical pancreatic tissues and 0–99.5% of pancreatic cancer cell lines. Invasion and migration assays demonstrated that CD166- pancreatic cancer cells showed stronger invasive and migratory activities than those of CD166+ cancer cells (p<0.05). On the other hand, CD166+ Panc-1 cells showed a significantly stronger colony formation activity than that of CD166- Panc-1 cells (p<0.05). *In vivo* analysis revealed that CD166+ cells elicited significantly greater tumor growth than that of CD166- cells (p<0.05) in both subcutaneous and orthotopic mouse tumor models. mRNA expression of the epithelial-mesenchymal transition activator Zeb1 was over-expressed in CD166- cells (p<0.001). Microarray analysis showed that TSPAN8 and BST2 were over-expressed in CD166+ cells, while BMP7 and Col6A1 were over-expressed in CD166- cells.

**Conclusions:**

CD166+ pancreatic cancer cells are strongly tumorigenic, while CD166- pancreatic cancer cells exhibit comparatively stronger invasive and migratory activities. These findings suggest that CD166 expression is related to different functions in pancreatic cancer cells.

## Introduction

Pancreatic cancer is one of the most lethal human malignancies, with a 5-year survival rate of less than 5% [1]. This poor outcome is largely because early diagnosis is uncommon and conventional therapeutics such as surgical resection, chemotherapy, and radiotherapy have limited efficacy [1], [2]. Therefore, new strategies are urgently needed for cancer therapy. Recently, the concept of cancer stem cells (CSCs) has received significant attention. CSCs comprise a very small population of cancer cells that have the ability to initiate and sustain tumor formation [3]. Consequently, targeted therapy of this small cell population in cancer might be more effective than current therapies including those for pancreatic cancer.

CD166, also known as activated leukocyte cell adhesion molecule (ALCAM), is a member of the immunoglobulin superfamily [4]. It is detectable in a wide variety of cell types, including epithelial cells, lymphoid cells, myeloid cells, fibroblasts, neuronal cells, hepatocytes, and bone marrow cells [5]. CD166 has been reported to be a marker for CSCs in colon cancer and prostate cancer, which indicates strong tumorigenicity [6], [7]. Moreover, its over-expression has been reported as an independent prognostic marker for some cancers [8]. On the other hand, inhibition of CD166 has been shown to enhance invasive and migratory activities in ovarian carcinoma and glioblastoma cells [9], [10]. In pancreatic cancer, there have been data reported on the relationship between CD166 expression and prognosis data, but it is still controversial [11]–[13].

In the present study, we evaluated the significance of CD166 expression in pancreatic cancer. We found that CD166+ pancreatic cancer cells exhibited stronger tumorigenicity than that of CD166- cells, whereas CD166- pancreatic cancer cells exhibited comparatively stronger invasive and migratory activities. These findings suggest that CD166 expression is related to different functions in pancreatic cancer cells.

## Materials and Methods

### Ethics statement

This study was approved by the Ethics Committee of Kyushu University (approval number: 24–222) and conducted according to the Ethical Guidelines for Human Genome/Gene Research enacted by the Japanese Government and the Helsinki Declaration. All patients provided signed informed consent approving the use of their tissues for unspecified research purposes. Mouse experiments were approved by the Ethics Committee of Kyushu University (approval number: A-24-262-0). The animals were housed under specific pathogen-free conditions.

### Patients and pancreatic tissues

Pancreatic cancer tissues were obtained from patients who underwent pancreatic resection at our institution. For immunohistochemistry, specimens were collected from 98 pancreatic cancer patients including 62 men and 36 women with a median age of 65.2 years (range: 36–81 years). The clinicopathological characteristics of the patients are described in Table 1. Overall survival was based on the date of the operation to the date of death or last follow-up. Prognoses were determined in September 2013. The median overall survival time was 16 months (range: 1–135 months). Sixty-six patients did not survive for the follow-up. Adjacent tissues to the specimens were evaluated histologically according to the criteria of the World Health Organization. The tumor stage was assessed according to the classification of the Union for International Cancer Control. As control tissues, we obtained 17 normal pancreatic tissue samples from intact pancreases that were resected for bile duct cancer, neuroendocrine tumor, or benign solid-pseudopapillary tumor. For flow cytometric analysis, pancreatic cancer tissues were collected from five patients, including three men and two women with a median age of 62.0 years (range = 37–80 years), which had been resected at our institution between July 2013 and November 2013.

**Table 1:** Clinicopathologic characteristics of the pancreatic cancer patients.

Median age		65.2 years (range, 36–81 years)
Sex	Male	62 (63.3%)
	Female	36 (36.7%)
Histological diagnosis	Invasive ductal adenocarcinoma	95 (96.9%)
	Adenosquamous carcinoma	3 (3.1%)
pT category	pT1	6 (6.1%)
	pT2	4 (4.1%)
	pT3	86 (87.8%)
	pT4	2 (2.0%)
pN category	pN0	22 (22.4%)
	pN1	76 (77.6%)
UICC stage	I	8 (8.2%)
	II	86 (87.87%)
	III	2 (2.0%)
	IV	2 (2.0%)
Histological grade	G1	15 (15.3%)
	G2	31 (31.6%)
	G3	46 (46.9%)
	others	6 (6.1%)
Pathological margin	Negative	62 (63.3%)
	Positive	36 (36.7%)

UICC, Union for International Cancer Control.

### Immunohistochemical procedures and evaluation

CD166 was detected using a mouse monoclonal antibody (clone MOG/07, 1:450; Novocastra, Newcastle upon Tyne, UK) by incubation overnight at 4°C. The EnVision system (Dako, Glostrup, Denmark) was used to visualize the immunostaining. Cells were considered positively immunostained when the membrane or cytoplasm was stained. Tissues without staining or weak and moderate staining intensities in <70% and <30% of cells, respectively, were considered as CD166^low^. CD166^high^ was assigned to tissues with weak and moderate staining intensities in ≥70% and ≥30% of cells, respectively. Sections were evaluated independently by two investigators without any knowledge of the clinical features of each case.

### Cells and culture conditions

Human pancreatic cells were dissociated from surgical pancreatic tissues using a Tumor Dissociation Kit (human) and gentleMACS Dissociator (Miltenyi Biotec, Bergisch-Gladbach, Germany) according to the manufacturer's instructions. Immediately after dissociation, the cells were analyzed by flow cytometry. In addition, we analyzed the following nine pancreatic cancer cell lines: BxPC3, CFPac-1, SW1990, AsPC1, and Capan-2 (American Type Culture Collection, Manassas, Va); Panc-1 (RIKEN, Tsukuba, Japan); MiaPaCa2, SUIT-2, and KP-2 (Health Science Research Resources Bank, Osaka, Japan). We also analyzed two normal pancreatic duct epithelial cell lines: a human primary normal pancreatic epithelial cell line, CS-PE (DS Pharma Biomedical Co., Osaka, Japan), and an immortalized pancreatic ductal epithelial cell line, HPDE6-E6E7 clone 6 (a gift from Dr. Ming-Sound Tsao, University of Toronto, Toronto, Canada). In addition, human pancreatic stellate cells (PSCs) were isolated from fresh pancreatic specimens using the outgrowth method [14]. The metastatic SUIT-2 cell line was previously established in our laboratory [15]. The same method was used to establish the metastatic Panc-1 cell line. Cells were maintained as described previously [16].

### Flow cytometric analysis and cell separation by immunoreactivity

Cells from subconfluent monolayer cultures were suspended in phosphate-buffered saline (PBS) and incubated with monoclonal anti-human ALCAM-phycoerythrin (PE) (R&D Systems, Minneapolis, MN), anti-human CD24-fluorescein isothiocyanate (FITC) (eBioscience Inc, San Diego, CA), anti-human CD44-FITC (MBL, Nagoya, Japan), and monoclonal anti-human CD133-FITC (Ancell Corp, Bayport, MN) antibodies. Mouse immunoglobulin G1 K isotype Control PE (eBioscience Inc) was used as a negative control. Mouse IgG1 K isotype Control PE and anti-CD11b-FITC (Miltenyi Biotec) were used to exclude mouse cells from analyses. Labeled cells were analyzed by a flow cytometer (EC800; Sony Biotechnology, Tokyo, Japan). For cell separation, we incubated magnetic microbeads conjugated with anti-PE reagent (Miltenyi Biotec) with labeled cells for 15 minutes at 4°C. Labeled cells were isolated by passing the suspension through an AutoMACS PRO separator (Miltenyi Biotec). Unlabeled cells were negatively selected and collected by the depletion method through the AutoMACS PRO separator.

### Matrigel invasion and migration assays

The invasiveness of pancreatic cancer cells was assessed by the number of cells that invaded through Matrigel (20 µg/well; BD Biosciences, Bedford, MA)-coated transwell chambers with 8-µm pores (BD Biosciences) as described previously [16], [17]. Cancer cells (5×10^4^ cells/0.25 mL) were seeded in the upper chambers and incubated for 24 h (Panc-1 cells) 48 h (SW1990 cells), or 72 h (SUIT-2 cells). Cancer cells that migrated to the lower surface of the membranes were fixed with 70% ethanol, stained with hematoxylin and eosin (H&E), and five random fields at 200× magnification were counted for Panc-1 and SW1990 cells or one center field at 100× magnification for SUIT-2 cells under a light microscope (BZ-9000E; Keyence, Osaka, Japan). The migration of pancreatic cancer cells was assessed using uncoated transwell inserts. The durations of incubation for the migration assay were 18 h for Panc-1 cells, 40 h for SW1990 cells, and 24 h for SUIT-2 cells. The results were expressed as the mean number of migrating cells in five random fields at 200× magnification. Each experimental condition was tested in triplicate, and three independent experiments were performed.

### Cell proliferation assay

Cell proliferation was evaluated by measuring the fluorescence intensity of propidium iodide (PI) as described previously [18]. Cells were seeded into six wells of a 24-well plate (Becton Dickinson Labware, Bedford, MA) at a density of 1×10^4^ cells/well. After incubation for the indicated times, PI (30 µmol/L) and digitonin (600 µmol/L) were added to each well to label nuclei with PI. The fluorescence intensity of PI, which corresponded to the total cell number, was measured using an Infinite F200 multimode reader (TECAN, Männedorf, Switzerland).

### Colony formation assay

Cells were seeded at a density of 1×10^3^ cells/well in Nunc 6-well cell-culture dishes (Thermo Fisher Scientific K.K., Yokohama, Japan) and incubated for 10 days. Then, the cells were stained with crystal violet, and the number of colonies was counted with the ChemiDoc XRS System (Bio-Rad Laboratories, Hercules, CA). All experiments were performed in triplicate dishes.

### Sphere formation assay

Cells were seeded at a density of 5×10^3^ cells/well in 6-well ultra-low attachment plates (Corning Inc., Corning, NY) and cultured in serum-free DMEM:Ham's F12 medium (Invitrogen, Carlsbad, CA) containing 20 ng/ml human recombinant epidermal growth factor (PeproTech, Rocky Hill, NJ), 10 ng/ml human recombinant fibroblast growth factor-2 (PeproTech), 1% Insulin-Transferrin-Selenium Solution (ITS-G), 200 U/ml penicillin, and 100 µg/ml streptomycin. After 12 days, the number of spheres consisting of >20 cells was counted and imaged under a light microscope. The experiment was performed twice.

### Adhesion assay

The adhesion assay was conducted as described in a previous study [12] with minor modifications. Cells were seeded at a density of 1 ×10^6^ cells/well in 24-well plates (Becton Dickinson Labware). After 60 min, the cells were washed with PBS to remove non-adherent cells. The adhesive cells were stained with crystal violet and counted under a light microscope. All conditions were tested in quadruplicate and the experiment was performed twice.

### In vivo experiments

Five-week-old female nude mice were implanted subcutaneously or orthotopically with cancer cells suspended in 100 µL PBS as described previously [19]. Three-dimensional diameters were measured to calculate the tumor volume. The mice were sacrificed on indicated day and the subcutaneous or orthotopic tumors were excised and weighed. For flow cytometric analysis, tumor cells were dissociated using the Tumor Dissociation Kit (human) and gentleMACS Dissociator.

### Silencing of CD166 expression by small interfering RNA

Cancer cells at approximately 90% confluence were transfected with CD166-7 (SI02780169) small interfering RNA (siRNA) (Qiagen, Tokyo, Japan) by electroporation using the Nucleofector System (Lonza, Bazel, Switzerland) according to the manufacturer's instructions. To verify the knockdown specificity, we used a control siRNA (Qiagen). The cells were used in subsequent experiments at 24–144 h after transfection.

### Quantitative RT-PCR (qRT-PCR)

Total RNA was extracted from cultured cells using a High Pure RNA Isolation Kit (Roche Diagnostics, Mannheim, Germany) and DNase I (Roche Diagnostics) treatment according to the manufacturer's instructions. qRT-PCR was performed using a QuantiTect SYBR Green Reverse Transcription-PCR kit (Qiagen) and the CFX96 Real-Time PCR System (Bio-Rad Laboratories). Primers were purchased from Takara Bio Inc (Tokyo, Japan). Primer sequences are shown in Table 2. Each reaction mixture was first incubated at 50°C for 30 min for reverse transcription to synthesize first-strand complementary DNA by priming the total RNA with a gene-specific primer. PCR was initiated by incubation at 95°C for 15 min to activate the polymerase, followed by 40 cycles of 95°C for 5 s, 60°C for 20 s, and 72°C for 30 s. Genes expression levels were calculated using a standard curve constructed with total RNA from Panc-1, SUIT-2, or specific PSCs. The expression levels of genes were normalized to those of β-actin as an internal control and expressed as the ratio of target gene expression to β-actin expression. All samples were run in triplicate, and each sample was analyzed at least twice. No detectable PCR products were amplified without prior reverse transcription. The accuracy and integrity of the PCR products were confirmed using an Agilent 2100 Bioanalyzer (Agilent Technologies Inc, Palo Alto, CA).

**Table 2:** Sequences of the oligonucleotide primers used in this study.

Primer	Forward sequence 5′-3′	Reverse sequence 5′-3′
CD166	tggcaatatcacatggtacaggaa	ccagggtggaagtcatggtatagag
E-cadherin	tcagcgtgtgtgactgtgaa	aggctgtgccttcctacaga
N-cadherin	cgaatggatgaaagacccatcc	gccactgccttcatagtcaaacact
Zeb1	catcttgagctgaatttgggtaaca	cctgaaatgacctgaagcatgaa
MMP2	ctcatcgcagatgcctggaa	ttcaggtaataggcacccttgaaga
TSPAN8	cctagcattagcaatatgggtacga	tgatgatggcacctacagcaa
BST2	ggatgcagagaaggcccaag	agtacttcttgtccgcgattctcac
BMP7	accagaggcaggcctgtaaga	ctcacagtagtaggcggcgtag
Col6A1	caccgactgcgctatcaagaa	gtcggtcaccacaatcaggtactta
β-actin	tggcacccagcacaatgaa	ctaagtcatagtccgcctagaagca

### Microarray analyses

We carried out microarray analyses of the CD166+ and CD166- cells derived from both Panc-1 and SW1990 cell lines. The quality of RNA samples was evaluated using the Agilent 2100 Bioanalyzer. A Human HT-12v4 Expression BeadChip (Illumina, San Diego, CA) was used for the analyses. Data were analyzed using BeadStudio software version 3.2.3 (Illumina).

### Statistical analysis

Values are expressed as the mean ± standard deviation. Comparisons between two groups were made using the Student t-test. Statistical significance was defined as p<0.05. The χ2 test was used to analyze the correlation between CD166 expression and clinicopathological characteristics observed in the immunohistochemical study. Survival was calculated by Kaplan-Meier analysis, and survival curves were compared using the log-rank test. All statistical analyses were performed using JMP 9.0.2 software (SAS Institute, Cary, NC).

## Results

### Cases of pancreatic cancer are often CD166^High^

**Figure 1:**
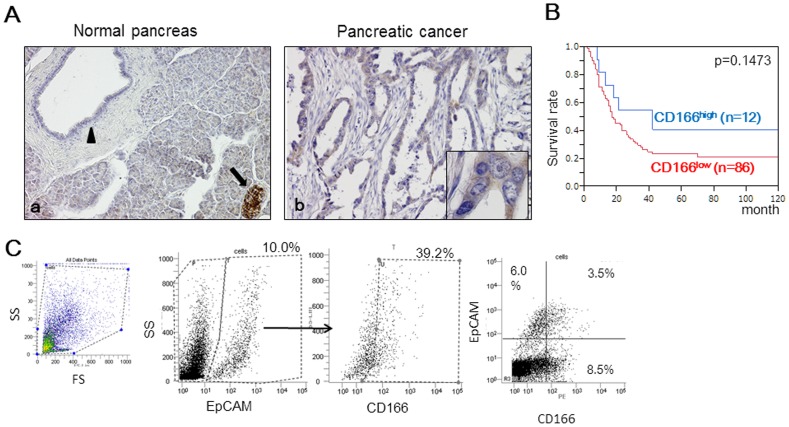
CD166 expression in human pancreatic tissues. (A) Immunohistochemical staining for CD166 was performed using resected pancreatic tissues. (a) In the normal pancreas, CD166 was expressed strongly in the membrane of islet cells (black arrows) and weakly in normal pancreatic ductal cells (black arrowhead). (b) In some pancreatic cancer tissues, cancer cells were positive for CD166. Original magnification: 200×. Insets: 600×. (B) Kaplan-Meier survival analysis revealed that the intensity of CD166 expression in pancreatic cancer was not correlated with prognosis (p = 0.1473). (C) Flow cytometric analysis of CD166 expression in cells separated based on EpCAM expression. The positive expression rate (%) is indicated for each marker.

Immunohistochemical staining for CD166 was performed using surgically resected pancreatic tissues (Figure 1A). Consistent with a previous study, we found strong CD166 staining in islet cells and moderate staining in nerves as positive controls [11]–[13]. In some cases, CD166 was expressed moderately in the acinar cells. In 17 normal pancreatic tissue samples, pancreatic ductal epithelial cells did not express CD166 or showed weak CD166 expression. All normal pancreatic tissues were identified as CD166^low^. In pancreatic cancer tissues, some cancer cells were stained moderately for CD166, and we identified 12.2% (12/98) of pancreatic cancer tissues as CD166^high^. The percentage of CD166^high^ pancreatic cancer tissues was significantly higher than that in normal pancreatic tissues (p = 0.0435). In pancreatic cancer tissues, we found that CD166 expression was associated with perineural invasion (p = 0.037, Table S1), but not prognosis using Kaplan–Meier survival analysis (p = 0.1473, Figure 1B).

We also evaluated CD166expression in pancreatic tissues by flow cytometry. In pancreatic cancer tissues, the CD166+ rate ranged from 15.2 to 45.3% (mean = 29.1%). Because CD166 is expressed in various types of cells [5], we used epithelial cell adhesion molecule (EpCAM), which is expressed exclusively in epithelia and epithelial-derived neoplasms, to exclude non-epithelial tissue from the analysis. Among EpCAM+ cells (10–56.5% of cells, mean = 20.6%), the positive rate of CD166 ranged from 33.8% to 70.2% (mean = 47.9%) (Figure 1C). There was no significant difference between CD166 and EpCAM positivity rates (p = 0.3488).

### CD166 Expression in human pancreatic cancer cell lines

**Figure 2:**
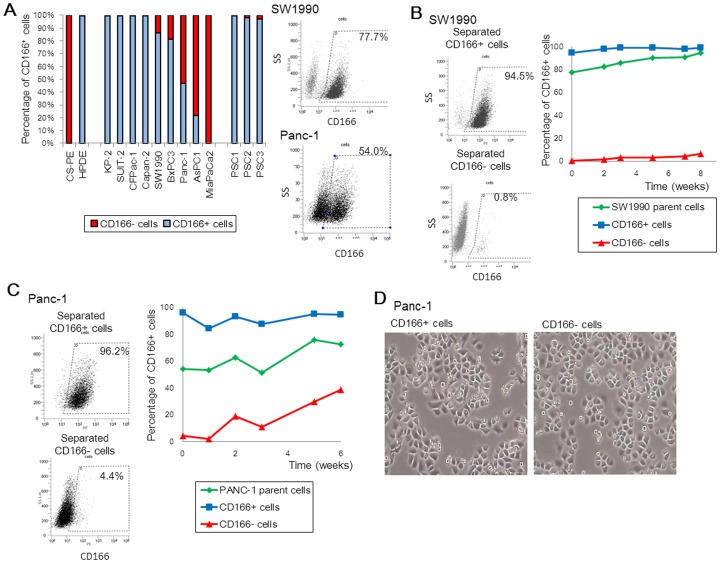
Analysis of CD166 expression in human pancreatic cancer cell lines. (A) CD166 positivity rates in two normal pancreatic duct epithelial cell lines, pancreatic cancer cell lines, and pancreatic stellate cells (PSCs). (B, C) SW1990 (B) and Panc-1 (C) cells (parental cells) were separated based on CD166 expression (CD166+ and CD166-) by an AutoMACS PRO separator. Changes in CD166 expression in parental and CD166+/− subpopulations were monitored frequently by flow cytometry over 6 weeks. (D) Morphology of Panc-1 cells separated based on CD166 expression. Original magnification: 40×.

Next, we analyzed the CD166+ rate in pancreatic cancer cell lines by flow cytometry, and found that CD166 was expressed in a wide range of cells (0–99.5%) (Figure 2A). Notably, no relationship was found between the CD166+ rate and malignant potentials, such as invasion, migration, and proliferation, which was in line with findings in a previous report (Table S2 and Figure S1) [20]. To further evaluate the significance of CD166 expression in pancreatic cancer, we analyzed SW1990 and Panc-1 cells among which 77.7–99.3% (mean = 86.4%) and 38.5–54.0% (mean = 46.9%) expressed CD166, respectively. After separation of the CD166+ and CD166- subpopulations, we checked CD166 expression weekly in parental, CD166+, and CD166- cells over a 6-week period (Figures 2B, C). The CD166+ rate did not significantly change in CD166+ cells, while that in both parental and CD166- cells increased gradually. Of note, we did not observe obvious morphological differences between CD166+ and CD166- Panc-1 cells (Figure 2D).

### CD166- cells show high invasive and migratory activities, whereas CD166+ cells show a stronger colony formation activity

**Figure 3:**
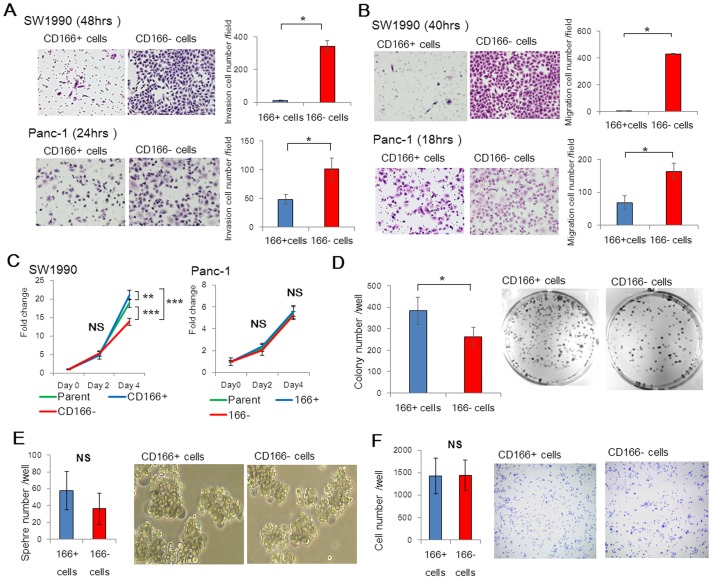
CD166- cells show high invasive and migratory activities. (A) Invasion assays of CD166+ and CD166- cells derived from SW1990 and Panc-1 cell lines were performed by culturing the cells on Matrigel-coated transwell inserts. After the indicated times, the cells on the lower membrane of the inserts were stained with H&E (representative images are shown) and quantified. (B) Migration assays of CD166+ and CD166- cells from SW1990 and Panc-1 cell lines were performed by culturing the cells on inserts. After the indicated times, the cells on the lower membrane of the inserts were stained with H&E (representative images are shown) and quantified. Original magnification: 200×. (C) Proliferation of CD166+ and CD166- cells derived from SW1990 and Panc-1 cell lines was measured at the indicated times post-initial seeding. (D) Quantification and representative images of the colony formation capacities of CD166+ and CD166- Panc-1 cells. (E) Quantification and representative images of the sphere formation capacities of CD166+ and CD166- Panc-1 cells. (F) Quantification and representative images of the adhesive capacities of CD166+ and CD166- Panc-1 cells. Original magnification: 40×. Data represent the mean ± SD; *, *p*<0.05; **, *p*<0.01; ***, *p*<0.0001; NS, not significant.

To explore the effects of CD166 in pancreatic cancer, various cancer-associated processes were tested in pancreatic cell lines that were separated based on CD166 expression. We compared the invasive and migratory abilities of CD166+ and CD166- cells derived from both SW1990 and Panc-1 cell lines. CD166- SW1990 and CD166- Panc-1 cells exhibited significantly greater cell invasion than that of their CD166+ cell counterparts (p<0.05; Figure 3A). In addition, CD166- cells exhibited a markedly increased migratory potential compared with that of CD166+ cells from both SW1990 and Panc-1 cell lines (p<0.05; Figure 3B). Using a proliferation assay, we found that CD166+ cells showed greater proliferative activity than that of CD166- cells from the SW1990 cell line (p<0.0001), but not CD166- cells from the Panc-1 cell line (Figure 3C). In the colony formation assay, CD166+ Panc-1 cells showed a significantly stronger colony formation activity than that of CD166- Panc-1 cells (p<0.05; Figure 3D). In sphere formation and adhesion assays, no differences were found between the capacities of CD166+ and CD166- Panc-1 cells (Figure 3E and 3F).

### CD166+ cells show strong tumorigenicity in mouse xenograft models

**Figure 4:**
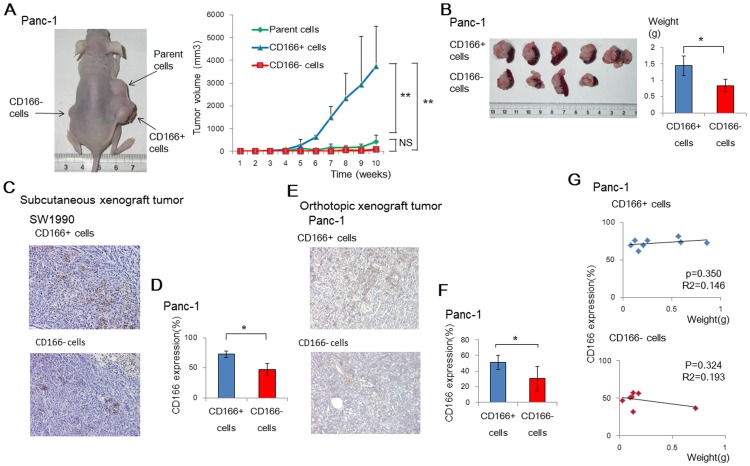
CD166+ cells show strong tumorigenicity in mouse xenograft models. (A) Mice were subcutaneously transplanted with parental, CD166+, and CD166- cells from the Panc-1 cell line (representative image), and tumor volumes were regularly measured for 7 weeks. (B) Mouse orthotopic tumor xenograft models were also generated from CD166+ and CD166- Panc-1 cells. Tumors were excised and wet weighed. (C, E) Immunohistochemical analysis of CD166 in subcutaneous (C) and orthotopic (E) tumors derived from CD166+ and CD166- SW1990 and Panc-1 cells. Original magnification: 100×. (D, F) CD166 expression was analyzed in subcutaneous (D) and orthotopic (F) tumors derived from CD166+ and CD166- cells by flow cytometry. (G) Analysis of the relationship between tumor weight and the positivity rate of CD166 cells in orthotopic tumors derived from CD166+ and CD166- Panc-1 cells. All graphs show the mean ± SD; *, *p*<0.05, **, *p*<0.01.

To evaluate the effects of CD166 on *in vivo* tumor growth, we subcutaneously transplanted CD166+ or CD166- cells into nude mice. We found that CD166+ cells had significantly greater tumorigenicity than that of CD166- cells derived from the Panc-1 cell line (p = 0.0082; Figure 4A and Table 3). Similarly, SW1990-derived CD166+ cells tended to generate larger tumors than those of CD166- cells, although the difference was not statistically significant (Figure S2 and Table S3). In mouse orthotopic xenograft models, tumors derived from CD166+ Panc-1 cells were heavier than those from CD166- Panc-1 cells (p<0.05; Figure 4B). Analysis of the subcutaneous and orthotopic xenograft models by flow cytometry showed that the CD166+ rate in tumors from CD166+ cells was significantly higher than that in tumors from CD166- cells, although immunohistochemistry did not show significant differences in CD166 expression (Figures 4C–F). There was also no significant relationship between the size of tumors and the CD166+ rate of cells (Figure 4G).

**Table 3:** Tumorigenic potential of CD166+/CD166- cells derived from the Panc-1 cell line.

		No. of mice with tumor formation		
		(Tumor volume>100 mm3)		
	No. of injected cells	Euthanized at 6 weeks	8 weeks	10 weeks
Parent	4×10 4	0/5	0/5	0/5
	2×10 5	0/5	2/5	4/5
	1×10 6	0/5	3/5	4/5
CD166+ cells	4×10 4	0/5	0/5	0/5
	2×10 5	0/5	0/5	0/5
	1×10 6	4/5	4/5	4/5
CD166- cells	4×10 4	0/5	0/5	0/5
	2×10 5	0/5	0/5	0/5
	1×10 6	0/5	0/5	1/5

### CD166- cells over-express the epithelial-mesenchymal transition (EMT) activator Zeb1

**Figure 5:**
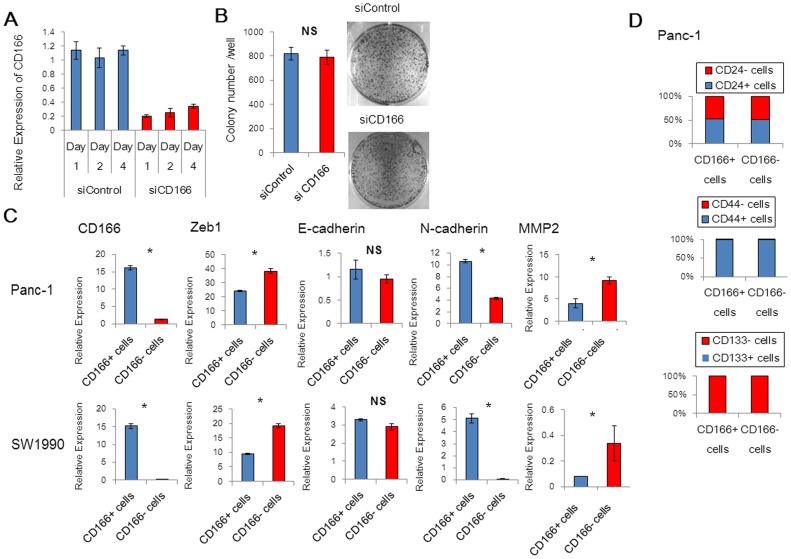
CD166- Cells Over-express the EMT Activator Zeb1. (A) qRT-PCR analysis of the mRNA levels of CD166 in SUIT-2 cells after RNA interference was performed at the indicated days post-transfection. Control (siControl) or CD166 silenced cells (siCD166) were analyzed by (B) colony formation assays at the indicated days post-transfection. (C) qRT-PCR analysis of EMT markers E-cadherin, N-cadherin, Zeb-1, and MMP2 in CD166+ and CD166- Panc-1 and SW1990 cells. (D) The relationships between expression of CD166 and CSC markers CD24, CD44, and CD133 in Panc-1 cells were analyzed by flow cytometry. Data represent the mean ± SD; *, *p*<0.05; NS, not significant.

Hong et al. reported that knockdown of CD166 by RNA interference has no effect on the growth or invasion of pancreatic cancer cells [12]. We also inhibited CD166 expression by RNA interference in the pancreatic cancer cell line SUIT-2. As a result, knockdown of CD166 did not affect invasion, migration, proliferation, or colony formation activities (Figures 5A, 5B, and S3A–C). To elucidate key factors underscoring the functional differences between CD166+ and CD166- cells, we next focused on the expression of markers for EMT and pancreatic CSCs. We evaluated the expression of EMT markers in SW1990 and Panc-1 cells using qRT-PCR. At the primary stage of EMT, cells lose expression of epithelial markers, express mesenchymal markers, and acquire motile and invasive properties [21]. The level of Zeb1 mRNA, an EMT activator, was greater in CD166- cells than that in CD166+ cells, although there was no difference in the mRNA levels of epithelial marker E-cadherin (Figure 5E). The level of N-cadherin mRNA, a mesenchymal marker, was higher in CD166+ cells than that in CD166- cells. Furthermore, the level of metalloproteinase 2 (MMP2) mRNA, which is related to cell invasiveness, was increased in CD166- cells compared with that in CD166+ cells [22]. Next, we analyzed the relationship between CD166 expression and CSC markers CD24, CD44, and CD133; however, we did not find any significant changes in their expression between CD166+ and CD166- cells derived from Panc-1 cells (Figure 5D) [23], [24].

### Microarray analysis shows over-expression of TSPAN8 and BST2 in CD166+ cells, while BMP7 and Col6A1 are over-expressed in CD166- cells

**Figure 6:**
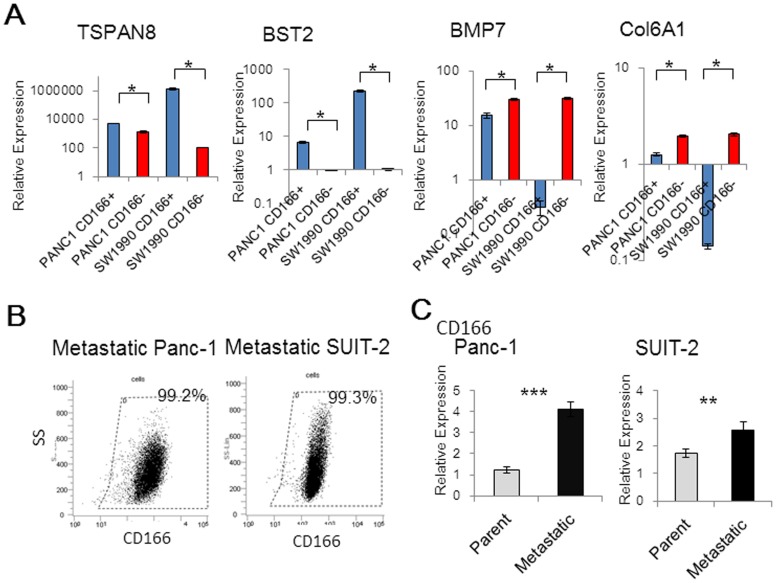
Over-expression of TSPAN8 and BST2 in CD166+ cells, and BMP7 and Col6A1 in CD166- cells. (A) qRT-PCR was used to analyze the mRNA levels of TSPAN8, BST2, BMP7, and Col6A1, which were found to be up-regulated by more than 2-fold in CD166+ and CD166- cells in comparisons of microarray data. (B) The positivity rate of CD166 in metastatic Panc-1 and metastatic SUIT-2 cell lines was measured by flow cytometry. (C) qRT-PCR analysis was also used to examine CD166 mRNA levels in the metastatic cell lines. Data represent the mean ± SD; *, *p*<0.05, **, *p*<0.01, ***, *p*<0.001.

To identify other key molecules involved in CD166 expression, we performed microarray analyses of CD166+ and CD166- cells from both Panc-1 and SW1990 cell lines. Comparisons of the microarray data identified 26 genes that were up-regulated by more than 2-fold in CD166+ cells (Table S4) and 11 genes that were up-regulated by more than 2-fold in CD166- cells (Table S5). Of these genes, we selected TSPAN8, BST2, BMP7, and Col6A1 for further analysis, because these genes have been reported to be involved in tumorigenicity or cancer cell invasion and migration. qRT-PCR analysis was then performed to validate microarray data (Figure 6A) [25]–[29]. To evaluate the effects of CD166 knockdown on the expression of these four genes, their mRNA levels were assessed in SUIT-2 cells after RNA interference. As a result, there were no significant changes in the expression levels of these genes (Figure S4).

To explore whether CD166 plays a role in metastasis, we used two previously established metastatic pancreatic cancer cell lines that were generated from liver metastases in nude mouse xenograft models [15]. These cell lines, metastatic Panc-1 and metastatic SUIT-2, showed a stronger liver metastatic potential compared with that of their parental cell lines. In the metastatic Panc-1 cell line, the CD166 expression rate was significantly increased compared with that of the parental cells (99.2% vs. 46.9%, Figure 6B). In the SUIT-2 cell line, most cells expressed CD166 in both parental SUIT-2 cells (99.3%) and metastatic SUIT-2 cells (99.4%). qRT-PCR analysis showed that the levels of CD166 mRNA in both metastatic Panc-1 and metastatic SUIT-2 cells were significantly greater than those in their parental cell lines (p<0.0001 and p = 0.0015 for Panc-1 and SUIT-2, respectively, Figure 6C).

## Discussion

In the present study, immunohistochemistry showed that CD166^high^ was often found in pancreatic cancer tissues compared to normal pancreatic tissues. Our flow cytometric analyses of resected pancreatic cancer tissues revealed that the percentage of CD166+ cells ranged from 33.8 to 70.2% among EpCAM+ cells, suggesting that CD166 expression was frequent in pancreatic cancers. However, the role of CD166 expression has not been clarified in pancreatic cancer. Previously, the roles of CD166 have been reported in several other types of cancer. However, the findings are controversial, because the cells expressing CD166 show strong tumorigenicities and inhibition of CD166 enhances invasive and migratory activities [6], [7], [9], [10].

Several studies have reported that CD166+ cancer cells in colon and prostate cancers might represent CSCs, because these cells exhibit strong *in vivo* tumorigenicity [6], [7]. In the present study, we found that the CD166+ pancreatic cancer cells had stronger tumorigenicity than that of their CD166- counterparts *in vivo*. We also found no differences in the expression rates of other pancreatic CSC candidate markers, CD24, CD44, and CD133 [23], [24], between CD166+ and CD166- cells. These results suggest that CD166 might be an independent marker of pancreatic CSCs. The present study revealed that CD166+ cells showed greater proliferation and colony formation abilities than those of CD166- cells *in vitro*. Although a greater sphere formation ability of CD166+ cancer cells has been reported in colon cancer, prostate cancer, and head and neck squamous cell carcinoma [6], [7], [30], our study showed that there was no significant difference in the sphere formation capacities of CD166+ and CD166- cells.

On the other hand, we showed that the CD166- population of pancreatic cancer cells had stronger invasive and migratory activities compared with those of the CD166+ population *in vitro*. The relationship between invasive/migratory abilities and CD166 expression has been previously reported in other types of cancer including endothelial-like yolk sac cells [31], epithelial ovarian carcinoma cells [9], and glioblastoma cells [10]. We also investigated the expression levels of EMT-associated genes in relation to the status of CD166 expression. We found that an EMT activator, Zeb1, was over-expressed in CD166- cells compared with that in CD166+ cells. However, there were no differences in morphology or expression of the epithelial marker E-cadherin. The role of CD166 might be related to pancreatic CSCs or EMT, but there are some controversial points.

To identify other key molecules involved in CD166 expression, our study showed that the level of MMP2 mRNA was greater in CD166- cells than that in CD166+ cells. Additionally, microarray analysis identified several genes that were differentially expressed in CD166+ and CD166- cells, including four genes, TSPAN8, BST2, BMP7, and ColA1, which are related to invasive and migratory activities or tumorigenicity [25]–[29]. Swart et al. and Hong et al. reported an association of CD166 with adhesiveness [5], [12]. Adhesiveness might cause the functional differences between CD166+ and CD166- cells; however, Hong et al. reported that knockdown of CD166 by RNA interference reduces cell adhesion but does not affect growth or invasion of pancreatic cancer cells [12]. Our study revealed that CD166+ cells showed strong proliferative activities, but there was no significant difference in the adhesive capabilities of CD166+ and CD166- cells. Further investigation is needed to clarify the functional difference between CD166+ and CD166- pancreatic cancer cells.

We examined the relationship between CD166 expression and metastatic potential in two previously established metastatic pancreatic cancer cell lines [15]. In these cell lines, the levels of CD166 mRNA expression were greater than those in their parental cell lines. Therefore, CD166 expression might be associated with the metastatic behavior of pancreatic cancer cells.

In conclusion, we have revealed that CD166+ pancreatic cancer cells are highly tumorigenic, whereas CD166- pancreatic cancer cells exhibit stronger invasive and migratory activities. Although further investigations are needed to uncover the mechanisms underlying these functional differences, this study demonstrates that CD166 expression is related to different functions in pancreatic cancer cells.

## Supporting Information

Figure S1 **Analysis of the relationships between CD166 positivity rates and malignant potential indicators (invasion, migration, and proliferation) in pancreatic cancer cell lines.**
(TIF)

Figure S2 **Mice were subcutaneously transplanted with parental, CD166+, and CD166− cells from the SW1990 cell line (representative image) and tumor volumes were regularly measured for 7 weeks.** Data represent the mean ± SD; NS, not significant.
(TIF)

Figure S3 **Effects of CD166 silencing by RNA interference on pancreatic cancer cell behavior.** Control (siControl) or CD166 silenced cells (siCD166) were analyzed by (A) invasion assays and (B) migration assays at the indicated days post-transfection. Original magnification: 200×. (C) Proliferation assay. Data represent the mean ± SD; NS, not significant.
(TIF)

Figure S4 **Effect of CD166 knockdown in SUIT-2 cells on the expression levels of TSPAN8, BST2, BMP7, and Col6A1.** SUIT-2 cells were transfected with CD166-targeting (siCD166) or control siRNA (siControl), and the expression levels of the four genes were assessed by qRT-PCR. Data represent the mean ± SD; ***, *p*<0.001; NS, not significant.
(TIF)

Table S1 **Relationships between CD166 expression and clinicopathological factors.**
(DOCX)

Table S2 **CD166 positivity rates and malignant potential indicators (invasion, migration, and proliferation) in each pancreatic cancer cell line as reported previously.**
(DOCX)

Table S3 **Tumorigenic potential of CD166+/− cells derived from the SW1990 cell line.**
(DOCX)

Table S4 **Differentially expressed genes by >2-fold in CD166+ cells.** (p<0.05).
(DOCX)

Table S5 **Differentially expressed genes by >2-fold in CD166- cells.** (p<0.05).
(DOCX)
