# Supplementary figures and images for: CD166/ALCAM Expression Is Characteristic of Tumorigenicity and Invasive and Migratory Activities of Pancreatic Cancer Cells
Source: PLoS One. 2014 Sep 15;9(9):e107247. doi: 10.1371/journal.pone.0107247 (PMC4164537; doi:10.1371/journal.pone.0107247)

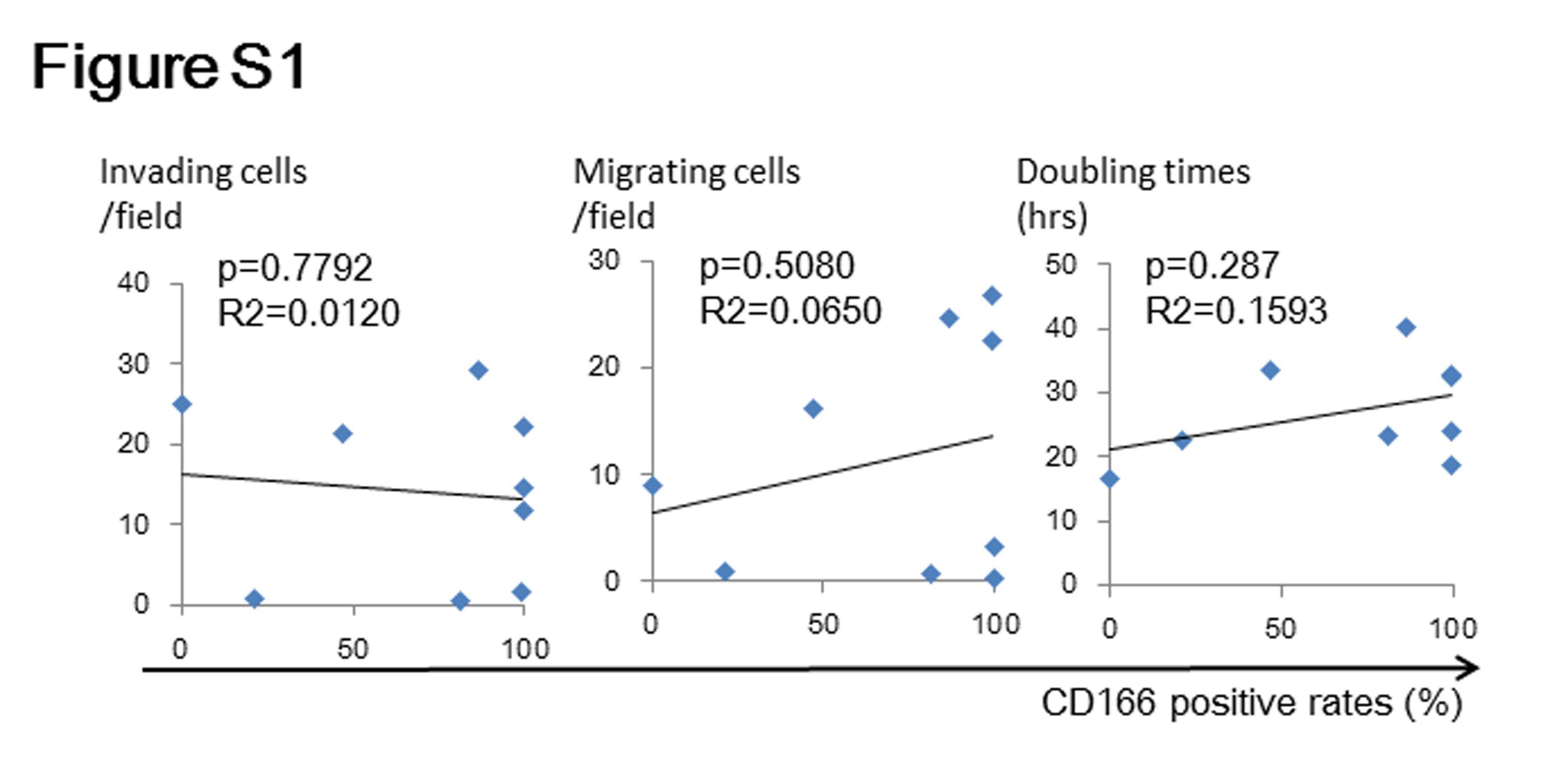

Supplement: Figure S1 — Analysis of the relationships between CD166 positivity rates and malignant potential indicators (invasion, migration, and proliferation) in pancreatic cancer cell lines. (TIF) [file pone.0107247.s001.tif]

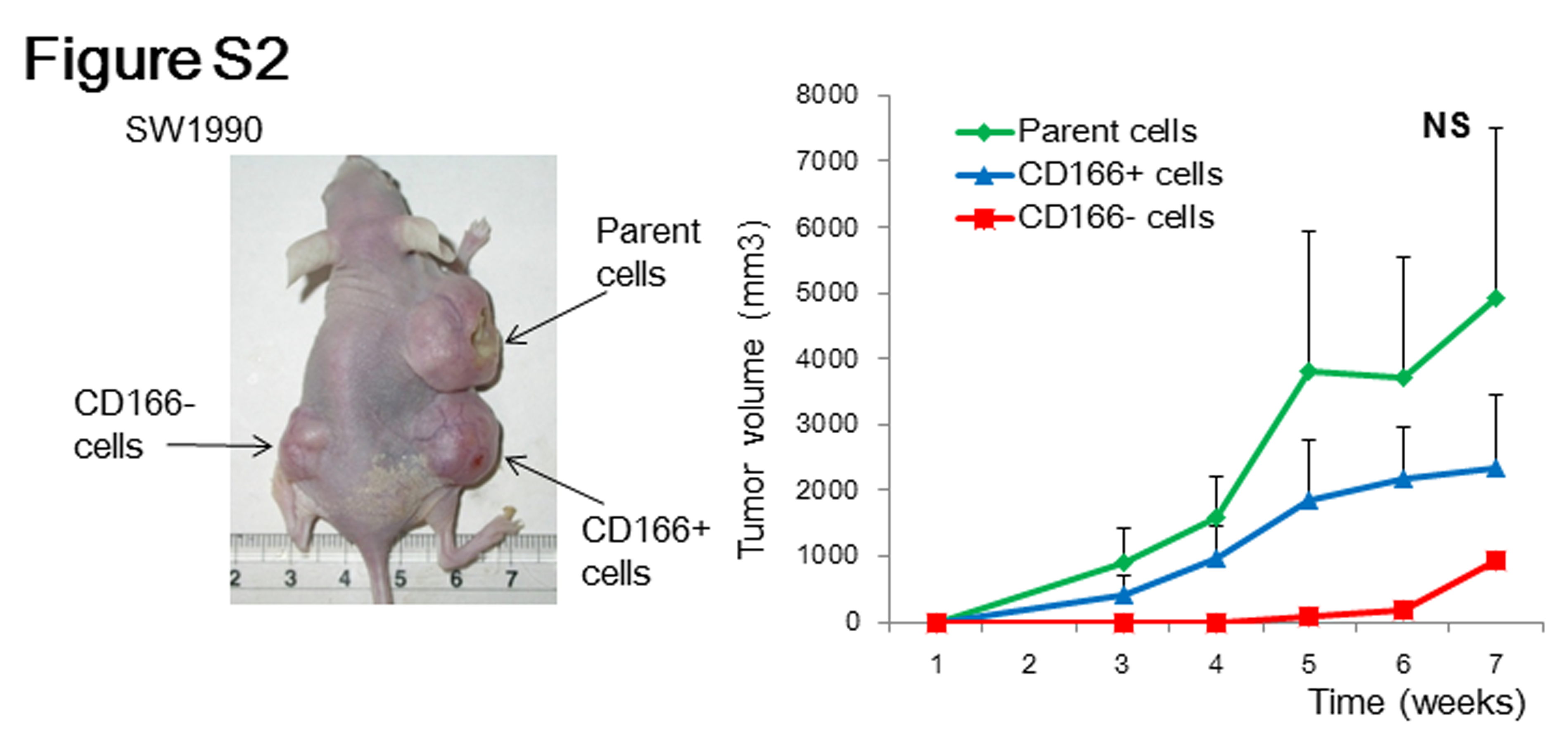

Supplement: Figure S2 — Mice were subcutaneously transplanted with parental, CD166+, and CD166− cells from the SW1990 cell line (representative image) and tumor volumes were regularly measured for 7 weeks. Data represent the mean ± SD; NS, not significant. (TIF) [file pone.0107247.s002.tif]

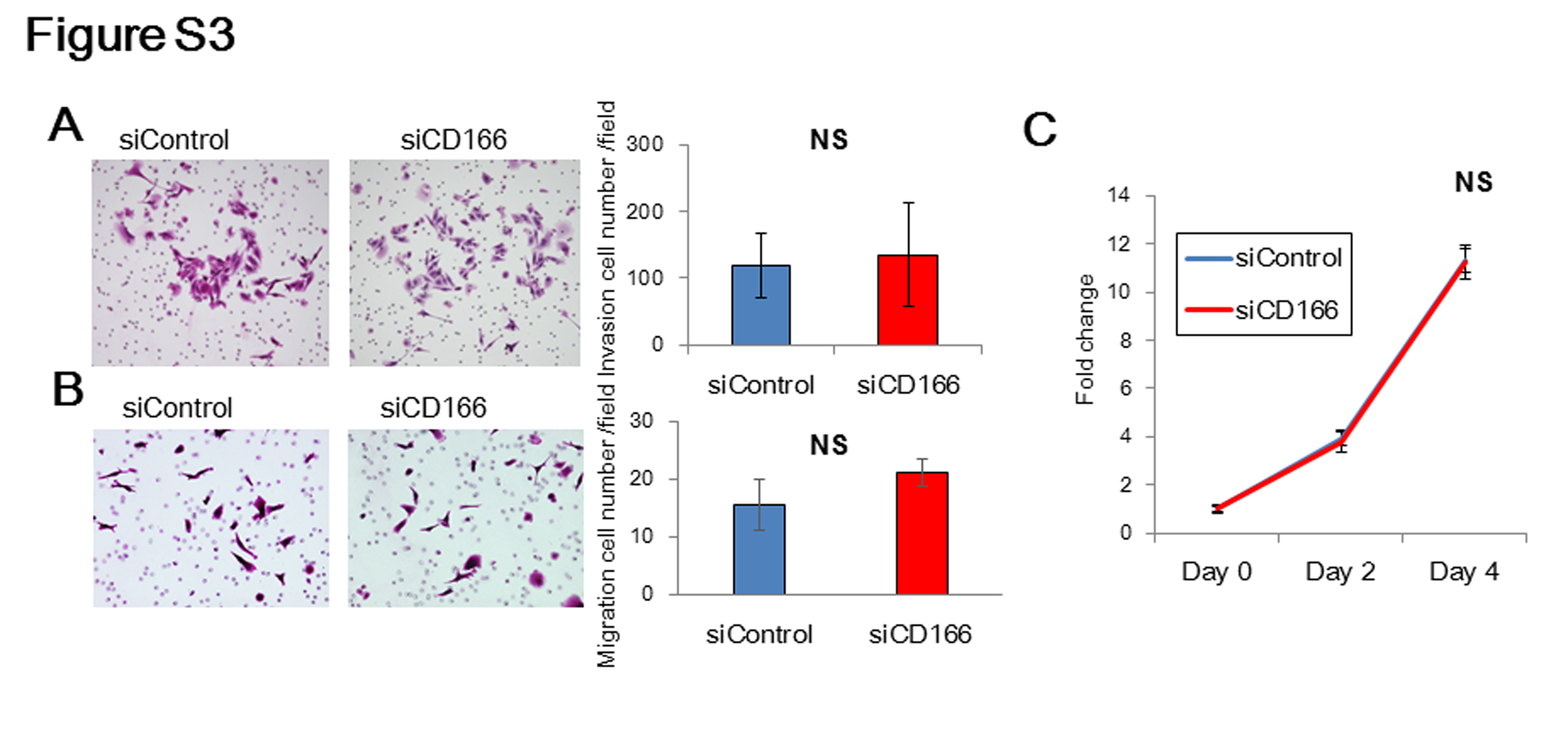

Supplement: Figure S3 — Effects of CD166 silencing by RNA interference on pancreatic cancer cell behavior. Control (siControl) or CD166 silenced cells (siCD166) were analyzed by (A) invasion assays and (B) migration assays at the indicated days post-transfection. Original magnification: 200×. (C) Proliferation assay. Data represent the mean ± SD; NS, not significant. (TIF) [file pone.0107247.s003.tif]

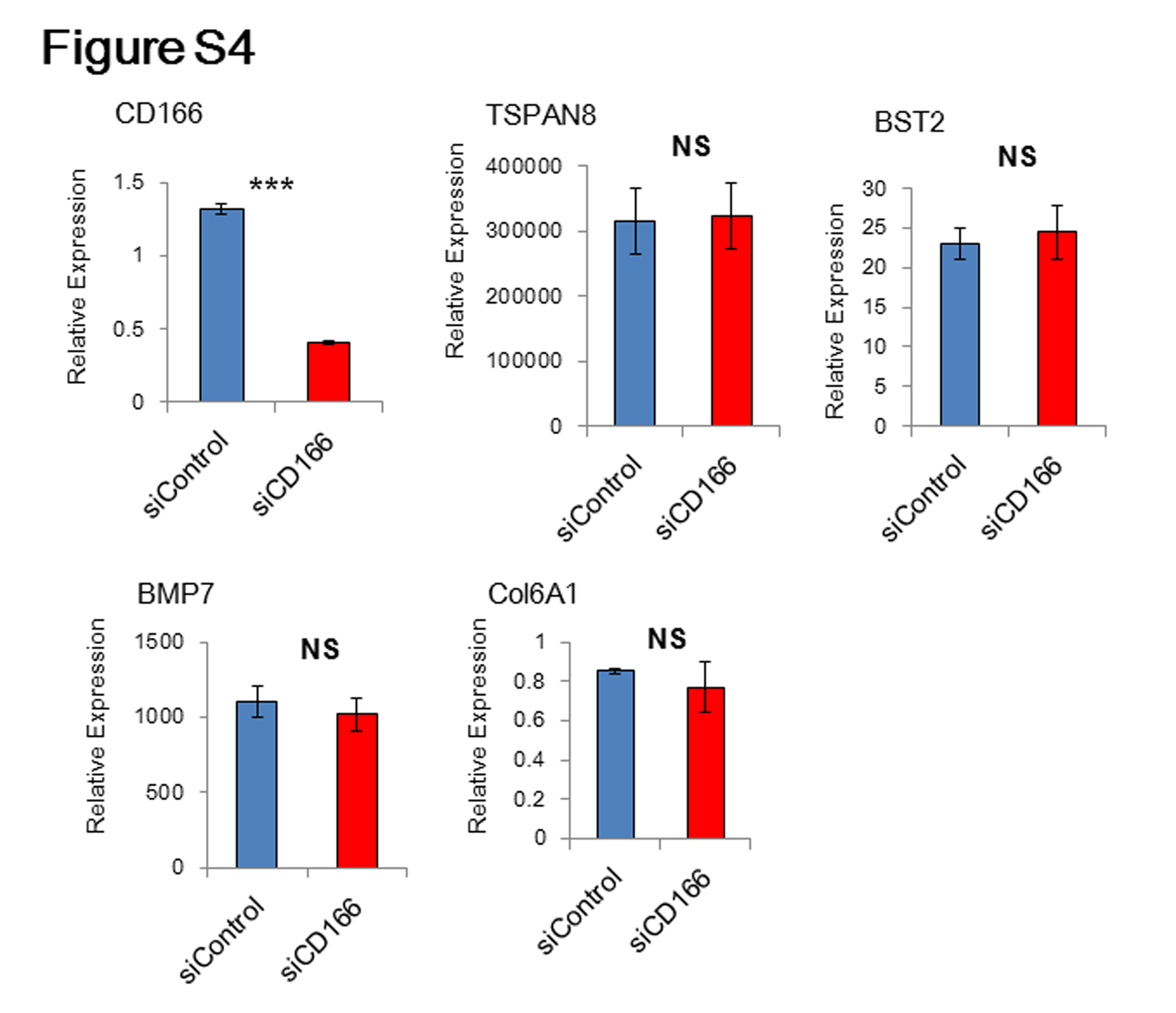

Supplement: Figure S4 — Effect of CD166 knockdown in SUIT-2 cells on the expression levels of TSPAN8, BST2, BMP7, and Col6A1. SUIT-2 cells were transfected with CD166-targeting (siCD166) or control siRNA (siControl), and the expression levels of the four genes were assessed by qRT-PCR. Data represent the mean ± SD; ***, p<0.001; NS, not significant. (TIF) [file pone.0107247.s004.tif]
