# Supplementary material for: CD166/ALCAM Expression Is Characteristic of Tumorigenicity and Invasive and Migratory Activities of Pancreatic Cancer Cells
Source: PLoS One. 2014 Sep 15;9(9):e107247. doi: 10.1371/journal.pone.0107247 (PMC4164537; doi:10.1371/journal.pone.0107247)
Supplement: Table S1 — Relationships between CD166 expression and clinicopathological factors. (DOCX) [file pone.0107247.s005.docx]

**Table S1.** Relationships between CD166 expression and clinicopathological factors.

|  |  | CD166 low, | CD166 high, |  |
| --- | --- | --- | --- | --- |
| Characteristics |  | n=86 (87.8%) | n=12 (12.2%) | p Value |
| Age | < 65 | 41 (47.7) | 4 (33.3) | 0.345 |
|  | ≥ 65 | 45 (52.3) | 8 (66.7) |  |
| pT category | pT1 / pT2 | 10 (11.6) | 0 (0.0) | 0.0963 |
|  | pT3 / pT4 | 76 (88.4) | 12 (100.0) |  |
| Histologic grade | G1 | 12 (14.0) | 3 (25.0) | 0.472 |
|  | G2 | 28 (32.6) | 3 (25.0) |  |
|  | G3 | 40 (46.5) | 6 (50.0) |  |
|  | others | 6 (7.0) | 0 (0.0) |  |
| Lymph node metastasis | No | 21 (24.4) | 1 (8.3) | 0.17 |
|  | Yes | 65 (75.6) | 11 (91.7) |  |
| UICC stage | I | 8 (9.3) | 0 (0.0) | 0.186 |
|  | II | 74 (86.1) | 12 (100.0) |  |
|  | III / IV | 4 (4.7) | 0 (0.0) |  |
| Perilymphatic invasion | No | 22 (25.9) | 2 (16.7) | 0.472 |
|  | Yes | 63 (74.1) | 10 (83.3) |  |
| Perivascular invasion | No | 31 (36.5) | 4 (33.3) | 0.831 |
|  | Yes | 54 (63.5) | 8 (66.7) |  |
| Perineural invasion | No | 15 (17.7) | 0 (0.0) | 0.037 |
|  | Yes | 70 (82.4) | 12 (100.0) |  |
| Pathological margin | Negative | 54 (63.5) | 7 (58.3) | 0.729 |
|  | Positive | 31 (36.5) | 5 (41.7) |  |

UICC, Union for International Cancer Control.
