# Supplementary material for: CD166/ALCAM Expression Is Characteristic of Tumorigenicity and Invasive and Migratory Activities of Pancreatic Cancer Cells
Source: PLoS One. 2014 Sep 15;9(9):e107247. doi: 10.1371/journal.pone.0107247 (PMC4164537; doi:10.1371/journal.pone.0107247)
Supplement: Table S2 — CD166 positivity rates and malignant potential indicators (invasion, migration, and proliferation) in each pancreatic cancer cell line as reported previously. (DOCX) [file pone.0107247.s006.docx]

**Table S2.** CD166 positivity rates and malignant potential indicators (invasion, migration, and proliferation) in each pancreatic cancer cell line as reported previously.

|  | KP-2 | SUIT-2 | CF-Pac1 | Capan-2 | SW1990 | BxPC3 | Panc-1 | AsPC1 | MiaPaca2 |
| --- | --- | --- | --- | --- | --- | --- | --- | --- | --- |
| CD166 positive rates (%) | 99.5 | 99.4 | 99.5 | 99.2 | 86.3 | 81.2 | 46.9 | 21.4 | 0 |
| Invading cells /field | 11.8 | 14.6 | 22.2 | 1.6 | 29.2 | 0.4 | 21.4 | 0.8 | 25 |
| Migrating cells /field | 0.2 | 22.5 | 3.2 | 26.7 | 24.7 | 0.6 | 16.1 | 1 | 9 |
| Doubling time (hrs) | 32.8 | 18.8 | 32.5 | 23.9 | 40.3 | 23.2 | 33.7 | 22.6 | 16.5 |
