# Supplementary material for: CD166/ALCAM Expression Is Characteristic of Tumorigenicity and Invasive and Migratory Activities of Pancreatic Cancer Cells
Source: PLoS One. 2014 Sep 15;9(9):e107247. doi: 10.1371/journal.pone.0107247 (PMC4164537; doi:10.1371/journal.pone.0107247)
Supplement: Table S3 — Tumorigenic potential of CD166+/− cells derived from the SW1990 cell line. (DOCX) [file pone.0107247.s007.docx]

**Table S3.** Tumorigenic potential of CD166+/- cells derived from the SW1990 cell line.

|  |  | No. of mice with tumor formation | | |
| --- | --- | --- | --- | --- |
|  |  | (Tumor volume>100mm3) | | |
|  | No. of injected cells | 3 weeks | 5 weeks | 7 weeks |
| Parent | 4x10 4 | 0/5 | 0/5 | 0/5 |
|  | 2x 10 5 | 2/5 | 3/5 | 3/5 |
|  | 1x 10 6 | 5/5 | 5/5 | 5/5 |
| CD166+ cells | 4x10 4 | 0/5 | 0/5 | 0/5 |
|  | 2x 10 5 | 0/5 | 1/5 | 1/5 |
|  | 1x 10 6 | 5/5 | 5/5 | 5/5 |
| CD166- cells | 4x10 4 | 0/5 | 0/5 | 0/5 |
|  | 2x 10 5 | 0/5 | 0/5 | 0/5 |
|  | 1x 10 6 | 0/5 | 0/5 | 1/5 |
