# Supplementary material for: CD166/ALCAM Expression Is Characteristic of Tumorigenicity and Invasive and Migratory Activities of Pancreatic Cancer Cells
Source: PLoS One. 2014 Sep 15;9(9):e107247. doi: 10.1371/journal.pone.0107247 (PMC4164537; doi:10.1371/journal.pone.0107247)
Supplement: Table S4 — Differentially expressed genes by >2-fold in CD166+ cells. (p<0.05). (DOCX) [file pone.0107247.s008.docx]

**Table S4.** Differentially expressed genes by >2-fold in CD166+ cells. (p<0.05)

| Gene symbol | CD166+ cells ave. | CD166- cells ave. | CD166+/ CD166- cells ratio |
| --- | --- | --- | --- |
| FOLR1 | 3905.8 | 87 | 44.89 |
| GAGE2B | 3720.7 | 97 | 38.36 |
| AKR1C3 | 3218 | 98.9 | 32.54 |
| KRT17 | 4598.9 | 153.2 | 30.02 |
| GAGE4 | 2582.4 | 86.4 | 29.89 |
| GAGE5 | 2296.9 | 91.3 | 25.16 |
| GAGE12G | 2451.5 | 99.2 | 24.71 |
| C4BPB | 2345.4 | 108 | 21.72 |
| OLR1 | 1630.7 | 89 | 18.32 |
| TSPAN8 | 1737.1 | 95.1 | 18.27 |
| BST2 | 1805.3 | 101.1 | 17.86 |
| MFAP5 | 1615.3 | 103 | 15.68 |
| KRT17P3 | 1460.3 | 118.9 | 12.28 |
| KRT7 | 1214 | 102.3 | 11.87 |
| LOC645638 | 939.3 | 121.2 | 7.75 |
| MOCOS | 728.1 | 106.3 | 6.85 |
| DSG2 | 756.8 | 118.9 | 6.37 |
| LGALS3BP | 700.6 | 163.1 | 4.30 |
| SLFN11 | 394.3 | 118.7 | 3.32 |
| MAPKAPK3 | 1210.6 | 395.2 | 3.06 |
| ALCAM(CD166) | 311.7 | 104.4 | 2.99 |
| PLEK2 | 297.3 | 114.2 | 2.60 |
| LOC148430 | 3854.4 | 1542.2 | 2.50 |
| LOC346887 | 228.5 | 94.6 | 2.42 |
| OCIAD2 | 937.8 | 394.9 | 2.37 |
| ACP6 | 346.8 | 155.6 | 2.23 |
