# Supplementary material for: CD166/ALCAM Expression Is Characteristic of Tumorigenicity and Invasive and Migratory Activities of Pancreatic Cancer Cells
Source: PLoS One. 2014 Sep 15;9(9):e107247. doi: 10.1371/journal.pone.0107247 (PMC4164537; doi:10.1371/journal.pone.0107247)
Supplement: Table S5 — Differentially expressed genes by >2-fold in CD166- cells. (p<0.05). (DOCX) [file pone.0107247.s009.docx]

**Table S5.** Differentially expressed genes by >2-fold in CD166- cells. (p<0.05)

| Gene symbol | CD166- cells ave. | CD166+ cells ave. | CD166-/ CD166+ cells ratio |
| --- | --- | --- | --- |
| TUBB2A | 2295.4 | 385.8 | 5.95 |
| TUBB2B | 5437.7 | 1350.7 | 4.03 |
| PRRX2 | 921.1 | 299.3 | 3.08 |
| BMP7 | 284 | 94.4 | 3.01 |
| LIPA | 2430.1 | 809.5 | 3.00 |
| PLK2 | 315.7 | 110.6 | 2.85 |
| COL6A1 | 277.3 | 109.4 | 2.53 |
| 5-Sep | 633.6 | 251.3 | 2.52 |
| HSPA12A | 234.3 | 103.7 | 2.26 |
| UNC84A | 1244.5 | 552.2 | 2.25 |
| ELOVL5 | 641.9 | 316.1 | 2.03 |
